# Hematological, Biochemical, and Performance Adaptations in Amateur Soccer Players Following a 4-Week Preseason Training Period

**DOI:** 10.3390/jfmk10030314

**Published:** 2025-08-14

**Authors:** Panagiotis Georgiadis, Pierros Thomakos, Ilias Smilios, Angeliki Papapanagiotou, Anastasia Evaggelatou, Gregory C. Bogdanis

**Affiliations:** 1 Department of Physical Education and Sport Science, Democritus University of Thrace, 69100 Komotini, Greece; panosgeo1994@gmail.com (P.G.); ismilios@phyed.duth.gr (I.S.); 2 School of Physical Education and Sports Science, National and Kapodistrian University of Athens, 17237 Dafne, Greece; pthom@phed.uoa.gr; 3 Department of Biological Chemistry, Medical School, National and Kapodistrian University of Athens, General Hospital of Athens “Laiko”, 11527 Athens, Greece; agpana@med.uoa.gr (A.P.); neva.11275@gmail.com (A.E.)

**Keywords:** football, aerobic fitness, countermovement jump, erythropoietin, uric acid, urea, MCV, MCH

## Abstract

**Background**: We examined changes in hematological, biochemical, and hormonal biomarkers, along with endurance and explosive performance indices, in amateur soccer players over a 4-week preseason period. **Methods**: Thirteen players (age: 19.7 ± 2.0 years; body mass: 73.0 ± 6.8 kg; height: 180 ± 0.1 cm; body fat: 8.6 ± 3.5%) were monitored during a 4-week preseason program, which included 21 training days, three friendly matches, and four days of rest. Before and after this period, endurance capacity was evaluated using the Yo-Yo IR1 test, and leg power was assessed using the CMJ. Blood samples were collected for three consecutive days in week 1 and after week 4 to assess hematological and biochemical parameters. Internal load during all weeks was assessed with session RPE (sRPE). **Results**: There was a 25.5% increase in Yo-Yo IR1 distance (2123 ± 413 vs. 1560 ± 356 m, *p* = 0.002), with the estimated VO_2_max and the speed associated with VO_2_max (vVO_2_max) improving by 8.7% (49.5 ± 3.0 to 54.2 ± 3.5 mL/kg/min, *p* = 0.002) and 5.3% (16.0 ± 0.7 to 16.9 ± 0.6 km/h, *p* = 0.002), respectively. In contrast, CMJ performance in weeks 2–4 declined by 13.4–21.0% relative to baseline, while sRPE peaked during week 3 (4011 ± 440 AU). Hematological variables were mostly stable except for small increases in MCV and MCH (1.5–1.8%, *p* < 0.001), while there were significant reductions in urea (12%), uric acid (6.2%), and erythropoietin (33%). **Conclusions**: A 4-week preseason program substantially improved aerobic capacity yet compromised leg power. Changes in biomarker profiles suggest that the training load maintained an appropriate balance between overload and recovery. These findings provide valuable guidance for coaches seeking to optimize training protocols while minimizing the risk of overtraining and preventing injuries during the competitive season.

## 1. Introduction

Soccer is a demanding intermittent sport that requires high aerobic and anaerobic capacity, muscular power, and agility to cover a total distance of 9–14 km per match, incorporating accelerations, decelerations, changes in direction, sprints, jumps, duels, and tackles [1,2,3,4,5]. During the 4–6 weeks of preseason training, strength and conditioning coaches aim to improve all aspects of sport-specific fitness to enhance in-season performance and reduce fatigue both within and between matches [6,7,8]. A high level of aerobic fitness forms the foundation of soccer-specific training as 80–90% of the total energy expended during a match is derived from aerobic metabolism [9]. In this context, examining changes in oxygen transport-related markers during the preseason—such as red blood cell count (RBC), mean corpuscular hemoglobin (MCH), and erythropoietin (EPO)—can provide valuable insights into the mechanisms underlying improvements in aerobic fitness.

High-intensity interval training (HIIT) is commonly used to improve aerobic fitness, and previous studies have shown that this type of training is associated with soccer match results [10,11]. However, implementing high volumes of HIIT and resistance training during the preseason period not only promotes adaptations but also causes fatigue [12]. Therefore, monitoring and controlling both external and internal loads is essential [13]. In this regard, the rating of perceived exertion (RPE), in the form of session RPE (sRPE), and the countermovement jump (CMJ) are commonly used to monitor internal load and detect possible neuromuscular overload, respectively, to prevent accumulated fatigue and overtraining during both the preseason and in-season periods [14,15,16,17].

The impact of preseason training load on the physiological state of players may also be assessed by measuring hematological and biochemical variables [18]. Limited studies using blood analyses have shown that hematological parameters, such as hemoglobin (Hb), hematocrit (Hct), and RBC, may increase during the preseason, contributing to the improvements seen in the aerobic fitness of the players [19]. However, there are few studies examining potential changes in specific oxygen transport-related parameters—such as RBC, mean corpuscular hemoglobin (MCH), and erythropoietin (EPO)—which could provide further insights into the mechanisms responsible for aerobic fitness improvements following periods of heavy aerobic training, such as the preparation phase in soccer. The endocrine system adapts both acutely and chronically to the training load [5,20]. For example, cortisol and testosterone levels, which are associated with catabolic and anabolic processes, respectively, are commonly used as markers of training-induced stress [5,15,19]. Several studies have shown that high training volumes, as well as a demanding match schedule, can decrease testosterone and increase cortisol levels, creating a hormonal profile indicative of overload [21]. Other blood markers, such as creatine kinase (CK), have been used to indirectly evaluate the degree of muscle microinjury within 2–5 days after a match [4,22,23,24,25,26,27]. Metabolic or oxidative stress markers, such as urea (U) and uric acid (UA), are also useful indicators of the metabolic stress imposed on players [5,22,23,24,25]. Elevated urea levels may reflect increased protein breakdown and metabolic stress resulting from intense exercise, while uric acid, a product of purine metabolism, also functions as an antioxidant [5]. Collectively, these markers provide insights into the balance between metabolic load and recovery, thereby helping to identify excessive stress or successful adaptation.

Although several studies have examined the hematological and biochemical responses to a soccer match [4,25,26,27], only few studies have been performed during the preseason period [19,28,29]. Thus, the aim of the present study was to examine changes in various blood biomarkers, including hematological, biochemical, and hormonal variables related to the heavy training load applied over a 4-week preseason period in amateur soccer players. In addition to blood parameters, endurance fitness and explosive performance indices—such as the aerobic Yo-Yo Intermittent Recovery Test Level 1 and the countermovement jump (CMJ)—were evaluated, while internal load was recorded using session-rated perceived exertion (sRPE). It was hypothesized that the intense preseason training program would improve aerobic fitness, causing at the same time overreaching, which would be evident by possible decreases in neuromuscular performance and increased internal load.

## 2. Materials and Methods

### 2.1. Experimental Approach

Thirteen amateur soccer players, all members of an amateur 1st-division team, participated in this study. All participants were familiar with the tests used as they had regularly performed them in previous years. Before the start of preseason training, players performed an aerobic fitness test on the field (Yo-Yo intermittent recovery test level 1) and a vertical jump test (CMJ). Preseason training comprised four weeks and included resistance training, HIIT, small- and medium-sided games, tactical games and three friendly matches (see Table 1). At the end of the intervention program, players rested for two days before post-training testing to avoid any effect from acute or residual fatigue or potentiation.

### 2.2. Participants

Sixteen players from an amateur team (1st division of Amateur league Athens–Greece) participated in the current study and signed a written informed consent form. None of the players received any medication or illegal nutritional supplements during the intervention. Players trained regularly in the past 3 years (5 sessions per week plus a match) and completed preseason training in the two seasons preceding the study. Players who sustained serious injuries in the previous season (>4 weeks out of training) were excluded. Also, younger players under the age of 18 and those who missed more than four training sessions (>20%) due to injuries or illness were excluded. Finally, data from thirteen players (age: 19.7 ± 2.0 years, body mass: 73.0 ± 6.8 kg, height: 180 ± 0.1 cm, body mass index: 22.6 ± 1.8, and body fat: 8.6 ± 3.5%) were analyzed after 4 weeks of pre-season training. All procedures were performed in accordance with the Declaration of Helsinki and approved by the Ethics Committee of the School P.E. and Sport Science of Democritus University of Thrace, Greece (Ref. number: 5351/18—date: 29 September 2022).

### 2.3. Procedure

**Figure 1:**
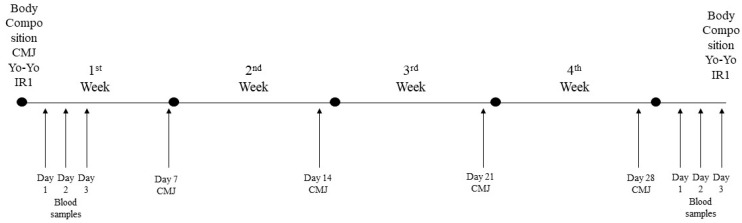
Timeline of measurements before, during, and after the 4-week intervention period.

The pre-season period lasted 4 weeks, which included 21 training days (3 of which had double training sessions), three friendly matches, and four days of rest. Six of the sessions included resistance training, followed by high-intensity aerobic training, while the afternoon sessions included small- and medium-sided games and tactical games. Each session lasted between 70 and 120 min. The warm-up mainly included balance and coordination exercises, and the cool-down included low-intensity running and flexibility exercises. Blood samples were collected in the morning (between 8:00 and 9:00) on the first three days at the start of the preseason and on the first three days following the intervention program, with players in a fasting state. Body composition, CMJ, and Yo-Yo Intermittent Recovery Level 1 (Yo-Yo IR 1) tests were performed at baseline and after the end of the 4-week training period. CMJ was also evaluated every week (Figure 1).

At the end of every session, the rating of perceived exertion (RPE) was recorded and session RPE (sRPE) was calculated as the product of the RPE and training duration. Food intake was not recorded, but the players were advised by an experienced sports nutritionist to follow a diet that was both quantitatively and qualitatively adequate according to their individual needs.

#### 2.3.1. Blood Sampling and Analysis

Blood samples were collected in the morning (between 8:00 and 9:00) on the first three days at the start of the preseason and on the first three days following the intervention program, with players in a fasting state. Ten milliliters of venous blood was drawn from the basilic vein with participants in a seated position. The samples were collected into two separate vials, placed in a portable refrigerator, and transported to the laboratory within 30 min of collection. One vial containing K_3_-EDTA was used to measure red blood cell count (RBC), white blood cell count (WBC), hemoglobin, hematocrit, and platelets. The other vial was centrifuged at 3000 rpm for 10 min, and the plasma was stored at −80 °C until assayed. Serum levels of erythropoietin were measured on an Elisa analyzer SEA C Brio2 (Calenzano, Italia) using a DRG EPO Immunoassay, which is a two-site enzyme-linked immunosorbent assay (ELISA), for the measurement of the biologically active 165-amino-acid-chain EPO. Erythropoietin measurements were performed in duplicate and in a blinded manner as the person analyzing them was unaware of the intervention and the labeling of the samples. Serum levels of creatinine, urea, uric acid, creatine kinase (CK), aspartate aminotransferase (SGOT), serum glutamic pyruvic aminotransferase (SGPT), total cholesterol, high-density lipoprotein cholesterol (HDL-C), low-density lipoprotein cholesterol (LDL-C), and triglycerides (Tg) were measured using a Cobas e801 biochemical analyzer (Basel, Switzerland) with reagents from Roche Hellas Company. Serum levels of cortisol, testosterone, and thyroid-stimulating hormone (TSH) were measured on a Hitachi Roche Cobas 8000 immunological analyzer (Basel, Switzerland) with reagents of the Roche Hellas company (Athens, Greece).

#### 2.3.2. Vertical Jump Test

The countermovement jump (CMJ) was used to evaluate the vertical jump ability and lower limb power, as well as neuromuscular fatigue, over the course of training. The depth of the countermovement was self-selected to avoid changes in coordination. The hands were placed on the hips throughout the whole movement, and athletes were directed to jump as high as possible and land close to the take-off point with the same body posture as that at take-off. They executed three maximal trials with a 2 min rest period, and the best result was selected for analysis. The CMJ was performed on an electronic mat (Chronojump—Bosco systems, Barcelona, Spain), and jump height was calculated using the following equation: jump height = (flight time^2^ × g)/8 [30].

#### 2.3.3. Aerobic Fitness

Aerobic fitness was evaluated by the Yo-Yo Intermittent Recovery Test Level 1. The test consisted of repeated 2 × 20 m runs back and forth, with progressively increasing speed controlled by audio beeps from a computer with speakers (Bangsbosport.com). Between each running bout, the subjects had a 10 s active rest period, consisting of 2 × 5 m of light jogging. When the subjects failed to reach the finishing line in two consecutive shuttle runs, the test was terminated. The total distance covered and the corresponding running speed (vV′O_2_max) were then recorded as the test results. V′O_2max_ was calculated from the follwing equation y = 0.0084 x + 36.4, where x is the distance covered in the test and y is V′O_2max_ in ml·kg^−1^·min^−1^ [31]. Heart rate was recorded for each player every 5 s using a wireless heart rate monitor worn around the chest (Suunto Oy, Vantaa, Finland).

#### 2.3.4. Rating of Perceived Exertion (RPE)

Rating of perceived exertion was obtained using the Borg scale (CR1-10) after 30 min at the end of every session to evaluate fatigue in the perceived effort of training and was recorded for every subject. Players were fully familiarized with the Borg scale, having used it regularly in their daily practice for at least two previous seasons. The researchers called each player individually, presented a visual scale, and recorded the player’s response. This value was multiplied by the session duration as a measure of internal load in arbitrary units [16].

### 2.4. Statistical Analysis

Data are presented as means ± standard deviations (SD). Statistical analysis was performed using appropriate software (SPSS, Version 29, Chicago, IL, USA). Paired *t*-tests were used to examine changes in Yo-Yo IR1 performance (distance, estimated V′O_2max_, vV′O_2max_). A two-way analysis of variance (ANOVA) with repeated measures on both factors (pre and post-training × 3 days) was used to examine changes in hematological, biochemical, and hormonal parameters. Changes in RPE and CMJ during the 4-week training period were compared using a one-way ANOVA. When a main effect or an interaction was found, differences between cell means were assessed using Tukey’s post hoc test. Cohen’s effect sizes (ESs) with 95% confidence intervals (95% CIs) were computed to determine the magnitude of every paired comparison and classified as follows (*d*): trivial (<0.2), small (0.2–0.6), moderate (0.6–1.2), large (1.2–2.0), and very large (2.0–4.0). The level of significance was set at *p* < 0.05. 

## 3. Results

### 3.1. Performance Assessments

#### 3.1.1. Body Composition

Body mass, body mass index (BMI), and percentage of body fat remained unchanged after the intervention program (pre vs. post: 73.0 ± 6.5 vs. 73.4 ± 6.8 kg, 22.6 ± 1.7 vs. 22.7 ± 1.8 kg m^2^, and 8.6 ± 3.4 vs. 8.9 ± 3.7%, respectively; *p* > 0.05).

#### 3.1.2. Aerobic Fitness

The distance covered during the Yo-Yo IR1 test increased by 25.5%, from 1560 ± 356 m (95% confidence interval [95% CI]: 1380 to 1740 m) to 2123 ± 413 m (95% CI: 1914 to 2332 m, *d*: 1.52, large, *p* = 0.002). Also, the estimated VO_2_max value improved by 8.7%, from 49.5 ± 3.0 (95% CI: 48.0 to 51.0) to 54.2 ± 3.5 mL/kg/min (95% CI: 52.4 to 56.0, *d*: 1.50, large, *p* = 0.002). The speed associated with VO_2_max (vVO_2_max) increased by 5.3%, from 16.0 ± 0.7 (95% CI: 15.6 to 16.4 km/h) to 16.9 ± 0.6 km/h (95% CI: 16.6 to 17.2 km/h, *d*: 1.44, large, *p* = 0.002).

#### 3.1.3. Vertical Jump

**Figure 2:**
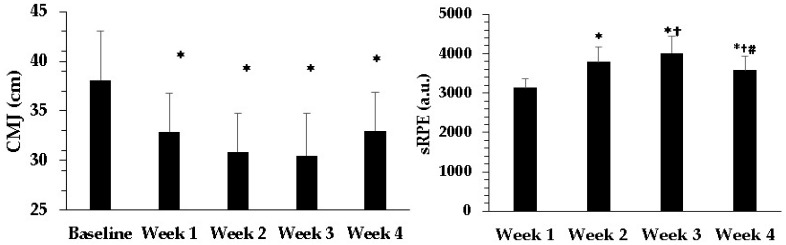
Countermovement jump performance (CMJ, **left panel**) and session rating of perceived exertion (sRPE, **right panel**) across four weeks of preseason training. *: *p* < 0.01 from week 1, †: *p* < 0.01 from week 1, and #: *p* < 0.01 from week 3.

CMJ decreased in week 1 of the intervention program compared with baseline, from 38.1 ± 4.9 cm (95% CI: 35.6 to 40.6 cm) to 32.9 ± 3.9 cm (95% CI: 30.9 to 34.9 cm, *p* < 0.001, *d* = 1.22 large), and remained lower thereafter (week 2: 30.9 ± 3.9 cm, 95% CI: 28.9 to 32.9 cm; week 3: 30.5 ± 4.3 cm, 95% CI: 28.3 to 32.7 cm; week 4 CMJ: 33.0 ± 3.9 cm, 95% CI: 31.0 to 35.0 cm, all *p* < 0.001, *d* = 1.20–1.72 larger compared to baseline). The decreases in CMJ ranged between 13.4 and 21% (see Figure 2, left panel).

#### 3.1.4. Session Rating of Perceived Exertion

Overall, the session RPE (sRPE) was high throughout the four-week preseason period. In week 1, sRPE was 3132 ± 233 AU. The values increased significantly in week 2 (3797 ± 370 AU; *p* < 0.001 vs. week 1) and peaked in week 3 (4011 ± 440 AU; *p* < 0.001 vs. week 1; *p* < 0.001 vs. week 2). In week 4, the sRPE decreased to 3585 ± 361 AU compared with weeks 2 and 3 (*p* < 0.001, see Figure 2, right panel).

### 3.2. Blood Analyses

#### 3.2.1. Hematological Parameters

The two-way ANOVA showed no significant main effects or interactions for all the hematological parameters measured, with the exception of MCV and MCH, which increased by about 1.5–1.8% (*p* < 0.001, see Table 2). There was also a tendency for an increase in platelet distribution (PDW, *p* = 0.075, ES = 0.53; see Table 2).

#### 3.2.2. Biochemical Parameters

Similarly, to the hematological parameters, most of the biochemical parameters remained unchanged after training. Only urea and uric acid showed decreases of 12% and 6.2%, respectively (Table 3). Also, the erythropoietin concentration decreased by 33% in week 4 (Table 3).

## 4. Discussion

The main finding of the present study is that four weeks of intense preseason training—including small- and medium-sided games, concurrent resistance strength training followed by HIIT, and a friendly match once per week—resulted in a substantial improvement in Yo-Yo IR1 test performance (26.5%), a 5.3% increase in vV′O_2_max, and an 8.7% increase in estimated V′O_2_max. However, this improvement in aerobic fitness was accompanied by a 13.4–21% decline in leg muscle power, as evidenced by the reduction in CMJ performance. The internal load, assessed by sRPE, mirrored the external load and peaked in week 3. Most hematological and biochemical parameters remained unchanged, with only MCV and MCH showing a slight increase, while urea, uric acid, and erythropoietin exhibited a decrease.

Improving aerobic fitness and the ability to perform repeated high-intensity efforts is a primary goal of the preseason [8]. Enhancements in fitness and adaptation to high training loads also reduce injury risk despite the substantial increase in external load during this period [32,33]. At baseline, our players were classified as moderately trained [31,34], and the magnitude of improvements in Yo-Yo IR1 performance (25.5%) and estimated V′O_2_max (8.7%) falls within the expected range for this phase [35]. In contrast, CMJ performance decreased significantly each week compared to baseline, indicating training overload and fatigue due to the high training load. This finding aligns with the results of Thomakos et al. [12], who showed that an increase in training load—resulting from the addition of concurrent resistance training and HIIT twice per week—improves Yo-Yo IR1 running performance but simultaneously causes a comparable decrease in CMJ. Morcillo et al. [14], in a study involving soccer players, reported a strong correlation between the percentage decrease in CMJ and increases in lactate and blood ammonia accumulation. This substantial drop in leg power output appears to depend on the training background of the individuals (i.e., less trained athletes experience a larger decline) and the contraction type used to assess fatigue, with stretch–shortening cycle activities being more affected [36].

The internal load, expressed as the sRPE, may be related to the players’ level of fatigue as it is calculated by multiplying the post-training rating of perceived exertion by training duration [16]. In the present study, sRPE peaked during the third week of training, which was characterized by the highest external load (see Table 1 and Table A1). In the same week, CMJ performance reached its lowest value. This decline in leg muscle power may reflect both the increased training load and cumulative fatigue from previous weeks [37]. The use of CMJ and sRPE as tools to monitor training progress is common in both running and soccer [15,38], and in the present study, they may reflect the magnitude of physiological load imposed on the players.

The blood biomarkers measured in the present study reveal another aspect of the adaptations occurring during a high-load training period such as the preseason [5]. It is well established that hematological measurements are linked to the development of aerobic capacity. During daily training lasting more than six weeks, increases in Hb and Hct indicate erythropoiesis, while increases in MCV and MCH reflect the formation of new red blood cells (RBCs) that are larger than mature RBCs, thereby raising the average MCV. Newer RBCs are not only larger but also contain more hemoglobin, resulting in increased MCH [39,40]. Additionally, endurance training may cause an expansion in the plasma volume, which can be as large as 10–20% [41]. Although plasma volume changes were not measured, which constitutes a recognized limitation of the present study, the similar Hct and Hb values at week 4 suggest that erythropoiesis matched plasma volume expansion [42]. A study of professional soccer players during three months of in-season training reported decreases in Hb and Hct accompanied by an increase in plasma volume [5,18]. In the present study, an initial expansion of plasma volume may have caused hemodilution, reducing the Hct and oxygen contents, which in turn may have stimulated erythropoietin synthesis and hematopoiesis [43,44]. As the RBC volume increased over the four weeks of training, erythropoietin levels normalized due to restored oxygen-carrying capacity and hematocrit, removing the stimulus for increased erythropoietin and potentially explaining its decrease in the fourth week of training (Table 3). However, part of the decrease in EPO concentration may be the possible expansion of the plasma volume, which was not assessed in the present study.

A decrease in urea and uric acid levels after exercise training (Table 3) typically reflects positive metabolic and renal adaptations, especially in the context of chronic, structured training, such as the endurance or mixed aerobic/anaerobic programs employed in the present study. Reduced urea levels may indicate decreased muscle breakdown, reflecting a reduction in catabolic processes, or could result from plasma volume expansion, which was not evaluated in the present study. The decline in uric acid following training may be attributed to reduced ATP degradation during intense exercise and/or decreased oxidative stress, leading to lower uric acid production or retention [45,46]. Studies investigating the effects of intense training periods in soccer players report similar alterations in hematological parameters depending on training load and duration [29,47,48,49]. The marginally significant increase in platelet distribution width (PDW%, *p* = 0.075, *d* = 0.53, medium effect; see Table 3) may reflect increased platelet turnover due to bone marrow stimulation by training, with newly produced platelets being larger and more active [50,51]. Additionally, this change may indicate an immune system response to the controlled inflammatory stimulus of exercise [52].

Hormonal and muscle damage markers, such as testosterone, cortisol, and creatine kinase, have been used as indicators of training overload and micro-damage, respectively [53]. Some studies have reported that T and C concentrations increased during the preseason in soccer [19,49]. In the present study there was no change in T and C values, possibly indicating successfully controlled overload and adaptation due to the periodization of training load achieved. In support of this, CK remained at relatively moderate levels (300–370 U/L) throughout the preseason period compared with data from a similar study that reported almost twofold higher CK values (599 U/L) in a single measurement at the end of the preseason period in semi-professional soccer players [54]. It is important to acknowledge that CK responses are modulated by both the athletes’ individual fitness levels and the magnitude of the training load applied. Therefore, such comparisons should be interpreted with caution to avoid confounding effects. The CK levels observed in the present study did not exceed those reported during the in-season period in soccer [25,27,54,55,56], possibly due to the controlled training load and match play time (see Table 1). Silva JR et al. [13] observed an increase in CK levels at the end of the soccer season in professional players due to the high number of matches played, which remained unchanged after the off-season period. The increased CK levels, indicating muscle overload and micro-damage, may partially explain the decreases in CMJ throughout the four preseason weeks [5].

The present study has some limitations. The preseason period where the measurements were performed was relatively short, which may resemble the time available in several cases (4 weeks) but is not the optimum time required for more effective adaptations (i.e., 6–8 weeks). The relatively small sample size (*n* = 13), the absence of a control group, and the use of estimated rather than directly measured VO_2_max values obtained via the Yo-Yo test may be considered additional limitations of the present study. However, as the study followed training of a team in real-life conditions, the number of players who completed all sessions may offer valuable information to practitioners. Another limitation is that we did not record the players’ diets during the experimental period. Instead, we advised the players to ensure that their diets were adequate both quantitatively and qualitatively by following the guidelines of an experienced sports nutritionist. Moreover, changes in strength were not measured, due to the refusal of the coach to perform any specific test, while recording of external load through GPS technology could not be carried out.

## 5. Conclusions

In conclusion, this study demonstrated that a short preseason period can lead to significant improvements in aerobic fitness and repeated high-intensity running performance. Hematological and biochemical data suggest a balance between overload and recovery, supporting positive physiological adaptations to intense training. A key finding was that leg power, as indicated by CMJ performance, decreased and remained suppressed throughout the preseason, likely due to the primary focus on aerobic conditioning. This knowledge may assist coaches in adjusting strength and power training based on players’ fatigue levels and adaptive capacity, as indicated by regular assessments of CMJ and sRPE. In addition, monitoring key hematological and biochemical biomarkers, alongside their interactions with variations in physical fitness, may provide valuable information for coaches and sport scientists aiming to optimize training strategies during the preseason—particularly for amateur soccer players who often experience extended periods of inactivity during the off-season. This approach may not only reduce the risk of overtraining but also contribute to a successful in-season period characterized by high fitness levels and a lower incidence of injuries.

## Figures and Tables

**Table 1:** Duration of each training element for each week of training (mean ± SD).

	Weeks						
Training Elements	1	2	3	4	Total	Mean	±SD
Warm-up (min)	60	60	60	60	240	60.0	0.0
Strength (min)	40	80	80	40	240	60.0	23.1
High-intensity running (min)	24	45	60	20	149	37.3	18.7
Small-sided games (min)	0	36	40	40	116	29.0	19.4
Medium-sided games (min)	116	48	26	40	230	57.5	40.0
Tactical games (min)	80	52	90	86	310	77.5	17.3
Match play (min)	0	45	45	55	145	36.3	24.6
Cool-down/stretching (min)	60	60	60	60	240	60.0	0.0
Sessions (count)	6	7	7	7	27		
Average session duration (min)	75	72	80	69			

**Table 2:** Hematological variables during the first week and after the fourth week of preseason training. Blood samples were drawn over the first three days of week 1 and after week 4. Data are shown as the mean ± SD. Bold indicates statistical significance (*p* < 0.05).

	Week 1			After Week 4					
Variable	Day 1	Day 2	Day 3	Day 1	Day 2	Day 3	Week 1 Average	Average After Week 4	Week 1 vs. After Week 4 *p* Level (Cohen’s d)
WBC (Κ/μL)	6.99 ± 1.77	6.72 ± 1.18	6.35 ± 1.25	6.11 ± 1.55	6.47 ± 1.56	6.51 ± 1.18	6.69 ± 1.41	6.37 ± 1.42	0.342 (0.23)
RBC (Μ/μL)	5.01 ± 0.37	5.05 ± 0.32	4.88 ± 0.34	4.93 ± 0.27	4.97 ± 0.32	4.80 ± 0.27	4.98 ± 0.34	4.90 ± 0.29	0.075 (0.25)
Hb (g/dL)	14.5 ± 1.4	14.4 ± 1.2	14.1 ± 1.2	14.4 ± 1.3	14.6 ± 1.3	14.2 ± 1.3	14.3 ± 1.2	14.4 ± 1.3	0.495 (0.06)
Hct (%)	42.4 ± 3.2	42.6 ± 2.7	41.1 ± 2.8	42.2 ± 3.3	42.8 ± 3.4	41.3 ± 3.0	42.1 ± 3.0	42.1 ± 3.2	0.996 (0.00)
MCV (fL)	85.1 ± 7.5	84.9 ± 7.6	84.7 ± 7.6	85.8 ± 7.5	86.4 ± 7.8	86.2 ± 7.7	84.9 ± 7.4	86.1 ± 7.5	<0.001 (0.17)
MCH (pg)	29.0 ± 3.0	28.9 ± 3.0	29.0 ± 3.1	29.4 ± 3.0	29.5 ± 3.1	29.6 ± 3.1	29.0 ± 3.0	29.5 ± 3.0	<0.001 (0.17)
MCHC (g/dL)	34.1 ± 1.0	34.0 ± 1.0	34.3 ± 1.1	34.2 ± 0.8	34.1 ± 0.9	34.3 ± 0.9	34.1 ± 1.0	34.2 ± 0.9	0.334 (0.11l)
RCDW (%)	12.9 ± 1.7	13.0 ± 1.8	13.0 ± 1.6	13.0 ± 1.3	13.0 ± 1.3	13.0 ± 1.3	12.9 ± 1.7	13.0 ± 1.3	0.764 (0.03)
PLT (Κ/μL)	286 ± 93	281 ± 78	265 ± 61	247 ± 36	259 ± 42	249 ± 35	278 ± 78	252 ± 37	0.164 (0.43)
MPV (fL)	10.5 ± 0.6	10.6 ± 0.6	10.8 ± 0.6	10.8 ± 0.6	10.8 ± 0.6	10.9 ± 0.6	10.6 ± 0.6	10.8 ± 0.6	0.052 (0.33)
PDW (%)	12.3 ± 1.1	12.2 ± 1.1	12.7 ± 1.5	13.0 ± 1.8	13.3 ± 1.5	13.3 ± 1.9	12.4 ± 1.2	13.2 ± 1.6	0.075 (0.53)
PCT (%)	0.29 ± 0.08	0.29 ± 0.07	0.28 ± 0.05	0.26 ± 0.04	0.28 ± 0.04	0.27 ± 0.04	0.29 ± 0.07	0.27 ± 0.04	0.458 (0.35)

WBC: white blood cell; RBC: red blood cell; Hb: hemoglobin; Hct: hematocrit; MCV: mean corpuscular volume of red blood cells; MCH: mean corpuscular hemoglobin; MCHC: mean corpuscular hemoglobin concentration; RCDW: red cell distribution width; PLT: platelet; MPV: mean platelet volume; PDW: platelet distribution width; PCT: Plateletcrit.

**Table 3:** Biochemical variables during the first week and after the fourth week of preseason training. Blood samples were drawn over the first three days of week 1 and after week 4. Data are shown as the mean ± SD. Bold indicates statistical significance (*p* < 0.05).

	Week 1			After Week 4					
Variable	Day 1	Day 2	Day 3	Day 1	Day 2	Day 3	Week 1 Average	Average After Week 4	Week 1 vs. After Week 4 *p* Level (Cohen’s d
U mg/dL)	35 ± 9	38 ± 9	37 ± 7	32 ± 1	31 ± 1	3 ± 7.0	37 ± 8	32 ± 10	0.043 (0.51)
Cr (mg/dL)	1.0 ± 0.1	1.0 ± 0.2	1.0 ± 0.2	1.0 ± 0.2	1.0 ± 0.2	1.0 ± 0.2	1.0 ± 0.2	1.0 ± 0.2	0.833 (0.00)
UA (mg/dL)	5.2 ± 0.9	5.6 ± 0.9	5.4 ± 1.3	5.2 ± 0.8	4.9 ± 1.0	5.0 ± 0.5	5.4 ± 1.0	5.0 ± 0.8	0.026 (0.36)
SGOT (U/L)	18 ± 4	21 ± 5	20 ± 6	20 ± 6	19 ± 7	18 ± 7	20 ± 5	19 ± 7	0.714 (0.12)
SGPT (U/L)	10 ± 1	10 ± 9	10 ± 6	10 ± 5	9 ± 6	9 ± 5	10 ± 8	9 ± 5	0.394 (0.20)
CK (U/L)	200 ± 122	309 ± 231	374 ± 196	362 ± 330	365 ± 383	325 ± 343	294 ± 197	351 ± 344	0.568 (0.20)
HDL (mg/dL)	53.1 ± 8.7	53.9 ± 9.5	51.1 ± 9.9	52.7 ± 8.8	51.5 ± 7.5	52.7 ± 7.1	52.7 ± 9.2	52.3 ± 7.6	0.743 (0.05)
LDL (mg/dL)	87.9 ± 27.8	89.7 ± 30.6	87.3 ± 28.0	85.3 ± 24.2	81.0 ± 29.8	84.0 ± 34.4	88.3 ± 28.1	83.4 ± 29.0	0.276 (0.17)
TC (mg/dL)	164 ± 30	164 ± 31	156 ± 31	157 ± 31	153 ± 37	158 ± 340	162 ± 30	156 ± 33	0.262 (0.18)
TG (mg/dL)	111 ± 54	103 ± 45	90 ± 40	95 ± 43	101 ± 44	109 ± 50	101 ± 46	102 ± 45	0.979 (0.00)
TSH (mIU/L)	2.00 ± 0.88	1.68 ± 0.67	1.87 ± 0.88	2.01 ± 0.97	1.92 ± 0.87	1.97 ± 0.70	1.85 ± 0.81	1.97 ± 0.83	0.343 (0.15)
T (µg/dL)	4.8 ± 1.3	4.98 ± 1.04	4.40 ± 1.41	4.67 ± 1.43	4.49 ± 1.60	4.82 ± 1.25	4.73 ± 1.25	4.66 ± 1.40	0.751 (0.05)
C (μg/dL)	7.7 ± 3.7	8.5 ± 3.1	8.0 ± 2.5	8.2 ± 3.0	7.8 ± 2.9	6.9 ± 2.7	8.1 ± 3.1	7.6 ± 2.9	0.545 (0.15)
EPO (IU)	6.3 ± 2.1	5.3 ± 4.4	8.1 ± 4.0	4.5 ± 2.4	4.0 ± 3.1	4.7 ± 3.1	6.6 ± 3.5	4.4 ± 2.8	0.038 (0.68)

U: urea; Cr: creatinine; UA: uric acid; SGOT: aspartate aminotransferase; SGPT: serum glutamic pyruvic aminotransferase; CK: creatine kinase; HDL: high-density lipoprotein; LDL: low-density lipoprotein; TC: total cholesterol; TG: triglycerides; TSH: thyroid-stimulating hormone; T: testosterone; C: cortisol; EPO: erythropoietin.

## Data Availability

The data are available upon request to the corresponding author.

## Abbreviations

ANOVA	Analysis of variance
C	Cortisol
CK	Creatine kinase
CMJ	Countermovement jump
Cr	Creatinine
EPO	Erythropoietin
GPS	Global positioning system
Hb	Hemoglobin
HDL	High-density lipoprotein cholesterol
Hct	Hematocrit
HIIT	High Intensity Interval Training
HR	Heart rate
LDL	Low-density lipoprotein cholesterol
MCV	Mean corpuscular volume
MCH	mean corpuscular hemoglobin
MCHC	mean corpuscular hemoglobin concentration
MPV	Mean platelets volume
PCT	Plateletcrit
PDW	Platelet distribution width
PLT	Platelets
PV	Plasma Volume
RBC	Red Blood Cells
RCDW	Red cells distribution width
RPE	Rating of perceived exertion
SGOT	aspartate aminotransferase
SGPT	Serum glutamic pyruvic aminotransferase
T	Testosterone
TC	Total cholesterol
TG	Triglycerides
TSH	Thyroid stimulating hormone
UA	Uric acid
V′O_2max_	Maximal oxygen uptake
vV′O_2max_	velocity of Maximal oxygen uptake
WBC	White blood cell count
Yo–Yo IR1	Yo–Yo Intermittent Recovery test, level 1

## Appendix A

**Table A1:** The intervention program for each of the four preseason weeks.

**1st Week**							
**Days**	**1**	**2**	**3**	**4**	**5**	**6**	**7**
Morning							
Afternoon	Warm up: 10 min MSG: 2 × 10 min, 4 min rest T-games: 15 min, 2 min rest Cool down: 10 min	Warm up: 10 min, MSG: 2 × 10 min, 3 min rest, T-games: 2 × 15 min, 3 min rest Cool down: 10 min	Warm up: 10 min, MSG: 3 × 8 min, 3 min rest, T-games 2 × 8 min, 2 min rest Cool down: 10 min	Warm up: 10 min, MSG: 3 × 10 min, 3 min rest, T-games: 10 min, 2 min rest Cool down: 10 min	Warm up: 10 min, Resistance training, HIR: 3 × 8 min, 80% vVO_2_max, 2 min rest Cool down: 10 min	Warm up: 10 min, MSG: 4 × 8 min, 4 min rest, T-games: 8 min, 2 min rest Cool down 10 min	Day off
**2nd Week**							
**Days**	**1**	**2**	**3**	**4**	**5**	**6**	**7**
Morning	Warm up: 10 min Resistance training HIR: 4 × 8 min, 80% vVO_2_max, 3 min rest, Cool down: 10 min			Warm up: 10 min Resistance training HIR: 3 × 8 min, 80% vVO_2_max, 3 min rest, Cool down: 10 min			
Afternoon	Warm up: 10 min MSG: 3 × 8 min, 4 min rest, T-game: 10 min Cool down: 10 min	Warm up: 10 min SSG: 4 × 5 min, 3 min rest, T-game: 2 × 8 min, 3 min rest Cool down: 10 min	Warm up: 10 min MSG: 3 × 8 min, 3 min rest T-games: 2 × 8 min, 3 min rest Cool down: 10 min		Warm up: 10 min SSG: 4 × 4 min, 4 min rest, T-game: 10 min Cool down: 10 min	Friendly Match 1	Day off
**3rd Week**							
**Days**	**1**	**2**	**3**	**4**	**5**	**6**	**7**
Morning	Warm up: 10 min Resistance training HIR: 5 × 6 min, 85% vVO_2_max, 3 min rest Cool down: 10 min		Warm up: 10 min Resistance training HIR: 5 × 6 min, 85% vVO_2_max, 3 min rest, Cool down: 10 min				
Afternoon		Warm up: 10 min SSG: 5 × 4 min, 3 min rest T-games: 2 × 15 min, 3 min rest Cool down 10 min	Warm up: 10 min MSG: 2 × 8 min, 3 min rest T-games: 2 × 15 min, 3 min rest Cool down 10 min	Warm up: 10 min SSG: 5 × 4 min, 3 min rest T-games: 2 × 10 min, 3 min rest Cool down 10 min	Warm up: 10 min MSG: 2 × 5 min, 3 min rest, T-games: 2 × 10 min, 3 min rest Cool down 10 min	Friendly Match 2	Day off
**4th Week**							
**Days**	**1**	**2**	**3**	**4**	**5**	**6**	**7**
Morning		Warm up: 10 min Resistance training HIR: 5 × 4 min, 90% vVO_2_max, 2 min rest Cool down 10 min					
Afternoon	Warm up: 10 min SSG: 5 × 4 min, 3 min rest T-game: 10 min Cool down 10 min	Warm up: 10 min T-games: 2 × 15 min, 5 min rest Cool down 10 min	Warm up: 10 min MSG: 3 × 8 min, 3 min rest T-games: 2 × 8 min, 3 min rest Cool down: 10 min	Warm up: 10 min SSG: 5 × 4 min, 3 min rest T-games: 2 × 10 min, 3 min rest Cool down: 10 min	Warm up: 10 min MSG: 2 × 8 min, 4 min rest T-game: 10 min Cool down: 10 min	Friendly Match 3	Day off

SSG: Small-Sided Games; MSG: Medium-Sided Games; T-Games: Tactical games; HIR: High Intensity Running; vVO_2max_: running velocity corresponding to maximum oxygen consumption.
